# Reduced Percentage of CD14^dim^CD16^+^SLAN^+^ Monocytes Producing TNF and IL-12 as an Immunological Sign of CLL Progression

**DOI:** 10.3390/ijms23063029

**Published:** 2022-03-11

**Authors:** Wioleta Kowalska, Michał Zarobkiewicz, Waldemar Tomczak, Justyna Woś, Izabela Morawska, Agnieszka Bojarska-Junak

**Affiliations:** 1 Department of Clinical Immunology, Medical University of Lublin, 20-093 Lublin, Poland; michal.zarobkiewicz@umlub.pl (M.Z.); justyna.wos@umlub.pl (J.W.); 51977@student.umlub.pl (I.M.); agnieszkabojarskajunak@umlub.pl (A.B.-J.); 2 Department of Haematooncology and Bone Marrow Transplantation, Medical University of Lublin, 20-080 Lublin, Poland; waldemar.tomczak@umlub.pl

**Keywords:** SLAN, monocytes, chronic lymphocytic leukemia

## Abstract

Monocytes are one of the least studied immune cells with a potentially important role in the pathogenesis of chronic lymphocytic leukemia (CLL). Nevertheless, data regarding the role of subpopulations of monocytes in the CLL microenvironment are still limited. For the very first time, this study presents an assessment of monocyte subsets divided according to SLAN and CD16 expression in CLL patients. The study involved 70 freshly diagnosed CLL patients and 35 healthy donors. Using flow cytometry, monocyte subpopulations were assessed among PBMCs. CD14^+^ monocytes can be divided into: “classical” (CD14^+^CD16^−^SLAN^−^), “intermediate” (CD14^+^CD16^+^SLAN^−^) and “non-classical” (CD14^dim^CD16^+^SLAN^+^). In our study, we noted an increased percentage of non-classical monocytes with intracellular expression of TNF and IL-12. On the other hand, among the intermediate monocytes, a significantly higher percentage of cells synthesizing anti-inflammatory IL-10 was detected. The percentage of CD14^dim^CD16^+^SLAN^+^ monocytes producing TNF and IL-12 decreased with the stage of CLL and inversely correlated with the expression of the prognostic factors ZAP-70 and CD38. Moreover, the percentage of CD14^dim^CD16^+^SLAN^+^ monocytes producing TNF and IL-12 was lower in CLL patients requiring treatment. This may indicate the beneficial effect of non-classical monocytes on the anti-tumor response.

## 1. Introduction

In CLL, one may observe several immune abnormalities, among which one can find changes in monocyte count and function. Initial studies have shown that the population of peripheral blood monocytes is heterogeneous due to the different expression of surface markers, namely CD14 and CD16 (FcγRIII) [1,2]. CD14 is a receptor for lipopolysaccharide (LPS) that binds to the Toll-like receptor 4 (TLR4) and is involved in the detection of the aforementioned LPS [3,4]. On the other hand, CD16 is a receptor for the IgG Fc fragment of immunoglobulin (FcγRIII), which in humans occurs in two variants: CD16a (FcɣRIIIa) and CD16b (FcɣRIIIb) [5]. The transmembrane isoform (FcɣRIIIa) is located on monocytes, macrophages and NK cells and is involved in the antibody-dependent cell-mediated cytotoxicity (ADCC) mechanism [6,7], while the FcɣRIIIb form is present on the surface of neutrophils [8]. In 2010, the International Consortium under the auspices of the International Union of Immunological Societies (IUIS) and the World Health Organization (WHO) developed a unified nomenclature of monocyte subpopulations [2]. The most abundant subpopulation is the one without the expression of the CD16 molecules, referred to as “classical” monocytes (CD14^++^CD16^−^), while all the remaining groups of monocytes express CD16 on their surface [2,9]. In the current project, we proved that, within CD16-positive monocytes, it is possible to distinguish cells with similar levels of expression of CD14 and CD16 molecules (CD14^+^CD16^+^). They were identified as the so-called intermediate monocytes. The second subpopulation of CD16-positive monocytes was found to be characterized by a very low expression of the CD14 molecule and a high expression of CD16 (CD14^low^/CD16^++^) [2,10]. In the following years, numerous attempts were made to understand the phenotypic and functional heterogeneity of each of the three discovered monocyte subpopulations [3,11].

In 2015, a divergence in the presence of the SLAN (6-sulfo LacNAc) molecule, an O-carbohydrate residue linked via 6-O-sulfotransferase to the glycoprotein ligand for P-selectin–PSGL-1, on the surface of CD16-positive cells, was observed [12]. In the same year, the nomenclature of CD16-positive monocytes was modified—the so-called “SLAN-dependent” monocytes were introduced. The CD16-positive monocyte population was divided into SLAN-positive non-classical monocytes (CD14^+^CD16^++^SLAN^+^) and SLAN-negative intermediate monocytes (CD14^++^CD16^+^SLAN^−^) [13]. The functional properties among individual subpopulations of CD16-positive SLAN-positive and CD16-positive SLAN-negative monocytes are still not fully understood. Understanding of the sophisticated interplay between aforementioned cells involved in antitumor response could be substantial for the diagnosis and successful treatment of CLL. Therefore, the aim of the study was to investigate the role of CD16-positive monocytes, with or without the expression of SLAN, as a part of the tumor microenvironment of CLL.

## 2. Results

### 2.1. The Percentage of the SLAN-Positive and SLAN-Negative CD16^+^ Monocytes Is Increased in CLL

**Figure 1:**
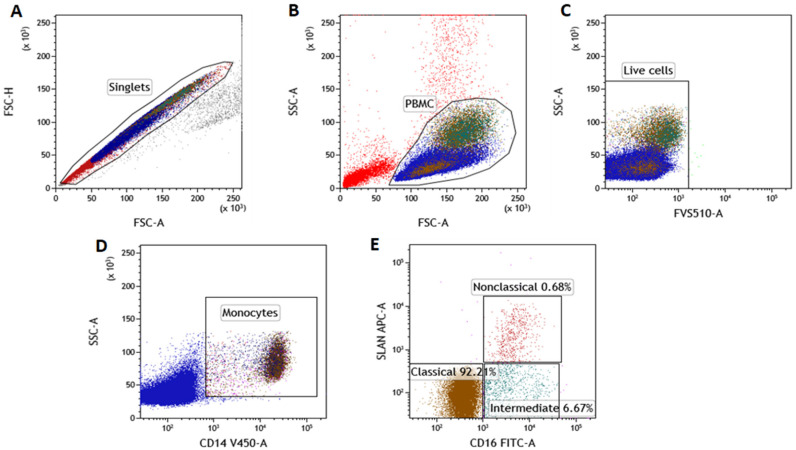
An example of the cytometric evaluation of SLAN-positive and SLAN-negative monocytes’ subpopulations from a CLL patient. (**A**) FSC-H vs. FSC-A dot plot of the doublets’ elimination (setting up gate covering the singlets population); (**B**) dot plots of SSC-A vs. FSC-A gating of PBMC cells; (**C**) dot-plot of the FVS510 vs. SSC exclusion of dead cells from further analysis. Live cells collected in the LIVE CELLS gate were used for further evaluation. (**D**) From the living cells, CD14^+^ cells (dot plot: SSC vs. CD14 V450; gating of Monocytes) were identified. The presence of the CD14 marker and scatter properties were the basis for the identification of monocytes. (**E**) SLAN APC vs. CD16 FITC dot plot—the differentiated expression of CD16 and SLAN molecules allowed the identification of classical (CD14^+^CD16^−^SLAN^−^), intermediate (CD14^+^CD16^+^SLAN^−^) and non-classical (CD14^dim^ CD16^+^SLAN^+^) monocytes.

Flow cytometry analysis was used to identify the subpopulation of monocytes with and without SLAN expression (Figure 1A–E). After the gating of a singlet, the selection of PBMC and the discrimination of live and dead cells (Figure 1A–C), among the CD14^+^ monocytes (Figure 1D), non-classical (CD14^dim^CD16^+^SLAN^+^), intermediate (CD14^+^CD16^+^SLAN^−^) and classical monocytes (CD14^+^CD16^−^SLAN^−^) were featured (Figure 1E).

**Figure 2:**
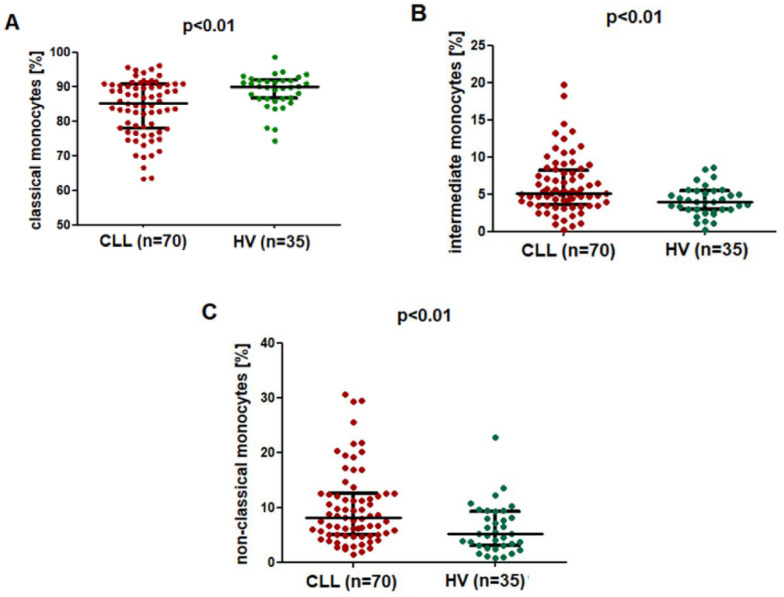
Comparison of the percentage of (**A**) classical CD14^+^CD16^−^SLAN^−^, (**B**) CD14^+^CD16^+^SLAN^−^ intermediate monocytes and (**C**) non-classical CD14^dim^CD16^+^SLAN^+^ monocytes in CLL patients and healthy volunteers. The solid line marks the median value, while the whiskers depict the interquartile range (IQR).

Classical monocytes were the most numerous among the CD14^+^ monocytes, both in the control group and the study group (Figure 2A). The percentage of classical (CD14^+^CD16^−^SLAN^−^) monocytes was significantly lower in CLL patients (median; IQR), at 85.37 (77.98–90.69%), as compared to the control group (median; IQR), with a percentage of 90.06 (86.69–92.00%) (*p* < 0.01) (Figure 2A). On the contrary, the percentage of CD14^+^CD16^+^SLAN^−^ intermediate monocytes was significantly higher in CLL patients, at (median; IQR) 5.18 (3.62–8.30%) as compared to 4.05 (2.96–5.48%) in healthy volunteers (*p* < 0.01) (Figure 2B). Similarly, in the group of CLL patients, a significantly higher percentage of CD14^dim^CD16^+^SLAN^+^ non-classical monocytes (median; IQR), namely 8.19 (5.11–12.61%), was found as compared to that of the control group (median; IQR), 5.20 (3.10–9.27%) (*p* < 0.01) (Figure 2C).

### 2.2. SLAN-Positive Monocytes Percentage Decreases with Disease Progression

**Figure 3:**
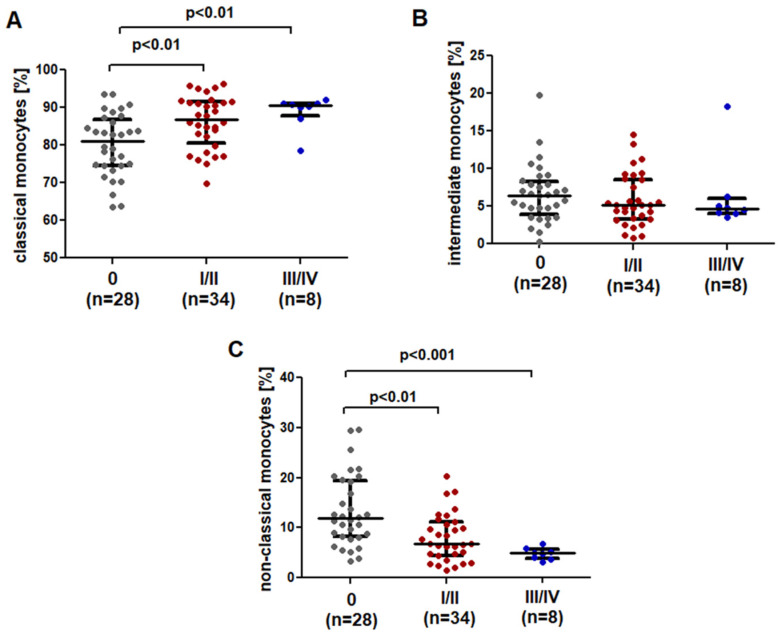
Comparison of the percentage of (**A**) classical, (**B**) intermediate and (**C**) non-classical monocytes in CLL patients at different stages of disease advancement. The solid line marks the median value. Ɪ represents the interquartile range (IQR).

A dependence between the percentage of CD14^+^CD16^−^SLAN^−^ classical monocytes and the clinical stage of CLL according to Rai was noted; the percentage of CD14^+^CD16^−^SLAN^−^ monocytes increased with disease progression, the lowest value was noted in the low-risk group (median (IQR) 81.14 (74.48–86.88)%). The percentage of classical monocytes was significantly lower in Rai stage 0, both compared to the intermediate risk group (median (IQR) 86.87 (80.43–91.55)%; *p* < 0.01) and the high-risk group (median (IQR) 90.53 (87.71–91.15)%; *p* < 0.01) (Figure 3A).

CLL patients in stage 0 showed a higher percentage of intermediate monocytes (CD14^+^CD16^+^SLAN^−^) (median (IQR) 6.54 (3.87–8.30)%) as compared to both patients with stage I/II (median (IQR) 5.12 (3.29–8.58)%) and III/IV (median (IQR) 4.63 (4.04–5.98)%), but the difference was not significant (Figure 3B).

CD14^dim^CD16^+^SLAN^+^ monocytes differed significantly between the patients of different risk groups. The percentage of non-classical monocytes decreased significantly with increased disease severity. Furthermore, in stage 0 according to Rai, the percentage of non-classical monocytes was significantly higher (11.87 (8.17–19.41%)) than in stage I/II (median (IQR) 6.77 (4.46–11.11)%) (*p* < 0.01) or stage III/IV (median (IQR) 4.92 (3.83–5.63)%) (*p* < 0.001) (Figure 3C).

### 2.3. SLAN-Positive Monocytes Are Less Numerous in CLL Patients with Negative Prognostic Factors

**Figure 4:**
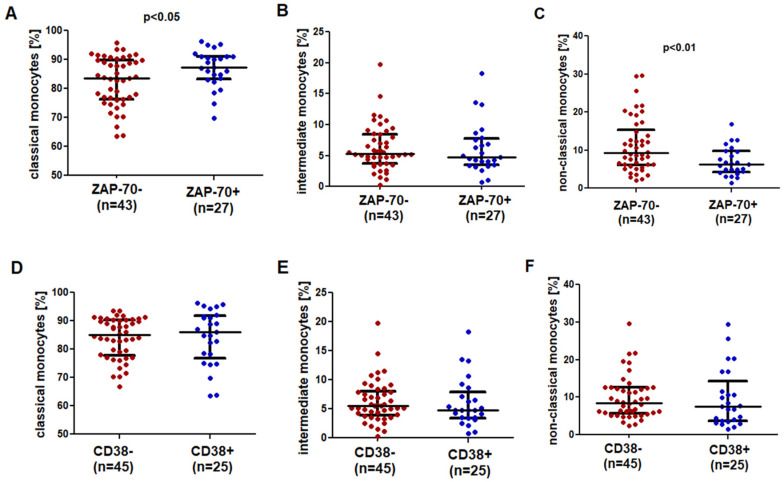
Comparison of the percentage of (**A**,**D**) classical, (**B**,**E**) intermediate and (**C**,**F**) non-classical monocytes in CLL patients ZAP-70-positive and ZAP-70-negative and CD38-positive and CD38-negative group. The solid line marks the median value. Ɪ represents the interquartile range (IQR).

A significantly higher percentage of classical monocytes was observed in ZAP-70-positive patients (Figure 4A). Intermediate monocytes did not differ between the ZAP-70-positive (median (IQR) 4.76 (3.45–7.72)%) and ZAP-70-negative (median (IQR) 5.34 (3.68–8.44)%) groups (Figure 4B). A significantly higher percentage of non-classical monocytes was noted in the ZAP-70-negative group (median (IQR) 9.22 (6.08–15.33)%) as compared to the ZAP-70-positive group (median (IQR) 6.25 (4.23–9.75)%; *p* < 0.01) (Figure 4C).

No significant differences in the percentage of classical, intermediate and non-classical monocytes between the groups of CD38-positive and CD38-negative patients were observed (Figure 4D–F).

**Figure 5:**
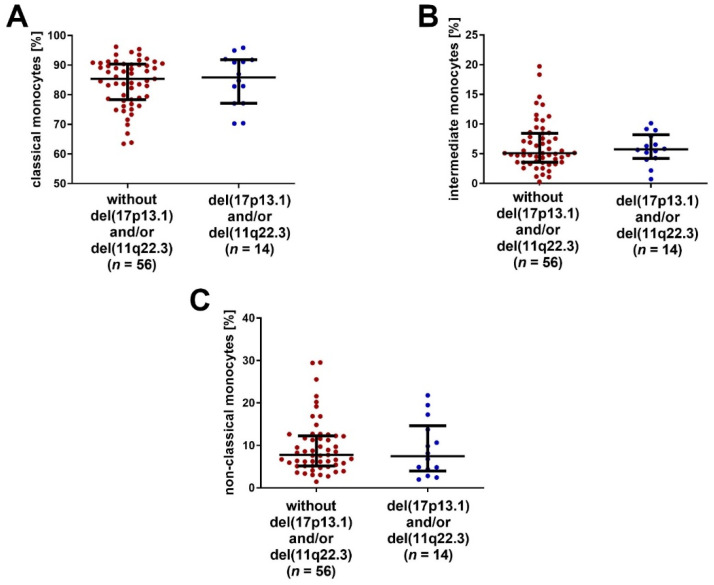
The percentage of monocytes (**A**) CD14^+^CD16^−^SLAN^−^, (**B**) CD14^+^CD16^+^SLAN^−^ and (**C**) CD14^dim^CD16^+^SLAN^+^ in the group of CLL patients with del (11q22.3) and/or del (17p13.1), and in the group of patients without these cytogenetic aberrations. The solid line marks the median value. Ɪ represents the interquartile range (IQR).

The percentages of classical, intermediate and non-classical monocytes were compared between the groups of patients with and without the negative cytogenetic aberrations del(11q22.3) and/or del(17p13.1). No significant differences between those groups were noted (Figure 5A–C).

No significant correlation was noted between the percentage of classical monocytes (CD14^+^CD16^−^SLAN^−^) and laboratory parameters (WBC, lymphocytosis, LDH activity, hemoglobin concentration and β2-microglobulin concentration) in the serum of CLL patients (*p* > 0.05). However, a positive correlation was observed between the percentage of intermediate monocytes and WBC (r = 0.299; *p* < 0.05) and lymphocytosis (r = 0.291; *p* < 0.05). There was also a positive correlation between the percentage of intermediate monocytes (CD14^+^CD16^+^SLAN^−^) and the percentage of CD5^+^CD19^+^ CLL cells (r = 0.318; *p* < 0.05). A negative correlation was observed between the percentage of non-classical monocytes (CD14^dim^CD16^+^SLAN^+^) and lymphocytosis (r = −0.365; *p* < 0.05), WBC (r = −0.373; *p* < 0.05) and the percentage of CD5^+^CD19^+^ leukemic lymphocytes (r = −0.304; *p* < 0.05).

### 2.4. SLAN-Positive Monocyte Subpopulation Was Decreased in CLL Patients Who Require Therapy

**Figure 6:**
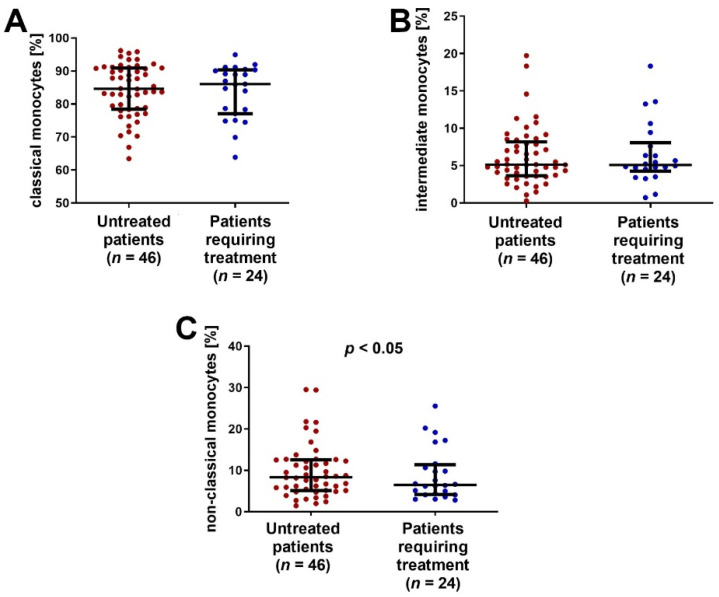
The percentage (**A**) classical, (**B**) intermediate and (**C**) non-classical monocytes in the group of CLL patients requiring treatment and in the group of patients not requiring antitumor treatment. The solid line marks the median value. Ɪ represents the interquartile range (IQR).

A significantly lower percentage of non-classical monocytes was observed in CLL patients who required cytostatic treatment (median (IQR) 6.49 (4.15–11.35)%) during the follow-up period (median (IQR) 8.37 (5.13–12.56)%; *p* < 0.05) (Figure 6C).

### 2.5. The Percentage of SLAN-Positive Monocyte Subpopulations and the Time-to-Treatment

**Figure 7:**
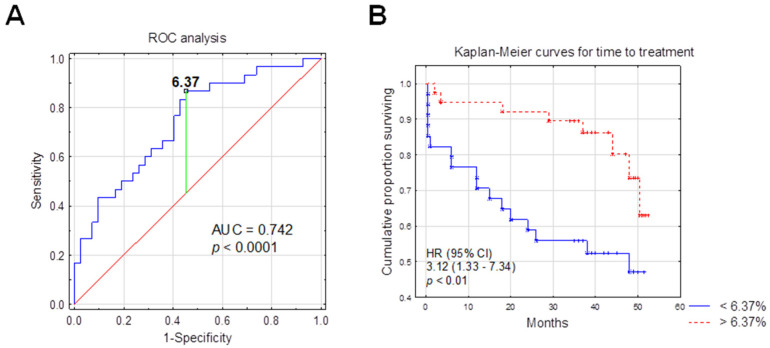
Low CD14^dim^CD16^+^SLAN^+^ percentage as a negative prognostic marker for the time-to-treatment (**A**) Graph of the ROC curve for the percentage of non-classical monocytes (CD14^dim^CD16^+^SLAN^+^) in ZAP-70-positive patients versus ZAP-70-negative CLL patients. ROC, receiver operating characteristic; AUC, area under the curve. ROC and AUC were used to calculate the most significant cut-off value of the non-classical monocytes (CD14^dim^CD16^+^SLAN^+^) percentage that best distinguished ZAP-70-positive and ZAP-70-negative CLL cases. (**B**) Kaplan–Meier curve comparing time-to-treatment-initiation (TTT) in groups of CLL patients with the percentage of non-classical CD14^dim^CD16^+^SLAN^+^ monocytes (≤6.37% and >6.37%). The division of CLL patients into two groups was made with reference to the cut-off point determined using the ROC curve analysis. HR, hazard ratio; CI, confidence interval.

Receiver operating characteristics (ROC) analysis was used to calculate the most significant cut-off value of the non-classical monocytes (CD14^dim^CD16^+^SLAN^+^) percentage that best distinguished ZAP-70-positive and ZAP-70-negative cases. Since ZAP-70 was previously proven to be one of the most powerful prognostic factors, it was used in ROC curve analysis. An area under the curve (AUC) was also estimated. The optimum threshold for the percentage of CD14^dim^CD16^+^SLAN^+^ associated with ZAP-70 above 20% was 6.37% (AUC, 0.742; sensitivity, 87%; specificity, 55%; 95% confidence interval (CI), 0.627–0.857; *p* < 0.0001) (Figure 7A).

The Kaplan–Meier analysis was used to assess the likelihood of the necessity to start cytostatic treatment (Figure 7B).

For the purposes of this study, the authors introduced the abbreviation nMo, which refers to a subpopulation of SLAN-positive non-classical monocytes (CD14^dim^CD16^+^SLAN^+^). In the nMo^high^ group (>6.37%), 21.62% of the patients required treatment during the follow-up period, while in the nMo^low^ group (≤6.37%), 44.48% of patients required treatment (Table 1).

There was a significant association between CD14^dim^CD16^+^SLAN^+^ monocytes percentage above 6.37% and longer TTT (hazard ratio (HR) = 3.12; 95% CI, 1.33–7.34; *p* < 0.01, median TTT: 39 months vs. 35 months in nMo^low^) (Figure 7, Table 2). Univariate Cox analysis selected ZAP-70, CD38 and B2M disruption as risk factors for shorter TTT, and these three parameters went for multivariate analysis. However, in multivariate analysis, a shorter TTT was not significantly associated with the CD14^dim^CD16^+^SLAN^+^ percentage (HR, 2.26; 95% CI, 0.98–5.23; *p* =  0.055) (Table 2).

The characteristics of patients from the non-classical low and high groups are presented in Table 1. In the nMo^high^ group, the percentage of CD19^+^/CD5^+^/ZAP-70^+^ was significantly lower than in the nMo^low^ group (*p* < 0.01). No significant differences were found in the percentage of CD19^+^/CD5^+^/CD38^+^ cells between nMo^high^ and nMo^low^ groups (Table 1). The analysis of laboratory parameters did not show any significant differences in platelets, hemoglobin levels, β2-microglobulin levels and serum LDH activity between the nMo^high^ and nMo^low^ groups. The nMo^low^ group was characterized by significantly higher monocytosis compared to the nMo^high^ group (*p* < 0.05) (Table 1).

### 2.6. Intracellular Expression of Cytokines in SLAN-Positive and SLAN-Negative Monocytes

**Figure 8:**
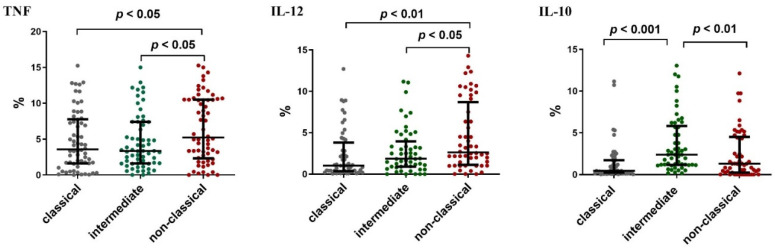
Percentage of classical (CD14^+^CD16^−^SLAN^−^), intermediate (CD14^+^CD16^+^SLAN^−^) and non-classical monocytes (CD14^dim^CD16^+^SLAN^+^) assessed for intracellular expression IL-10, TNF and IL-12 in “ex vivo” conditions. The solid line marks the median value. Ɪ represents the *interquartile range* (IQR).

In order to understand the functional activity of individual monocyte subpopulations, the ability of these cells to produce selected pro- and anti-inflammatory cytokines was assessed immediately after blood sampling from CLL patients (ex vivo). Intracellular expression of IL-10, TNF and IL-12 were assessed. Intermediate monocytes were skewed towards IL-10 production while non-classical ones were skewed towards TNF and IL-12. (Figure 8). The highest percentage of TNF+ cells was observed among the non-classical monocytes (median (IQR) 5.23 (2.32–10.51)%), while the lowest among the intermediate ones (median (IQR) 3.36 (1.62–7.40)%; *p* < 0.05). Additionally, the percentage of non-classical monocytes with intracellular expression of IL-12 (CD14^dim^CD16^+^SLAN^+^IL-12^+^) was significantly higher (median (IQR) 2.63 (1.13–8.69)%) than that of intermediate monocytes (median (IQR) 1.87 (0.91–3.96)%) and classical monocytes (median (IQR) 1.04 (0.36–3.82)%; *p* < 0.05) (Figure 8).

**Figure 9:**
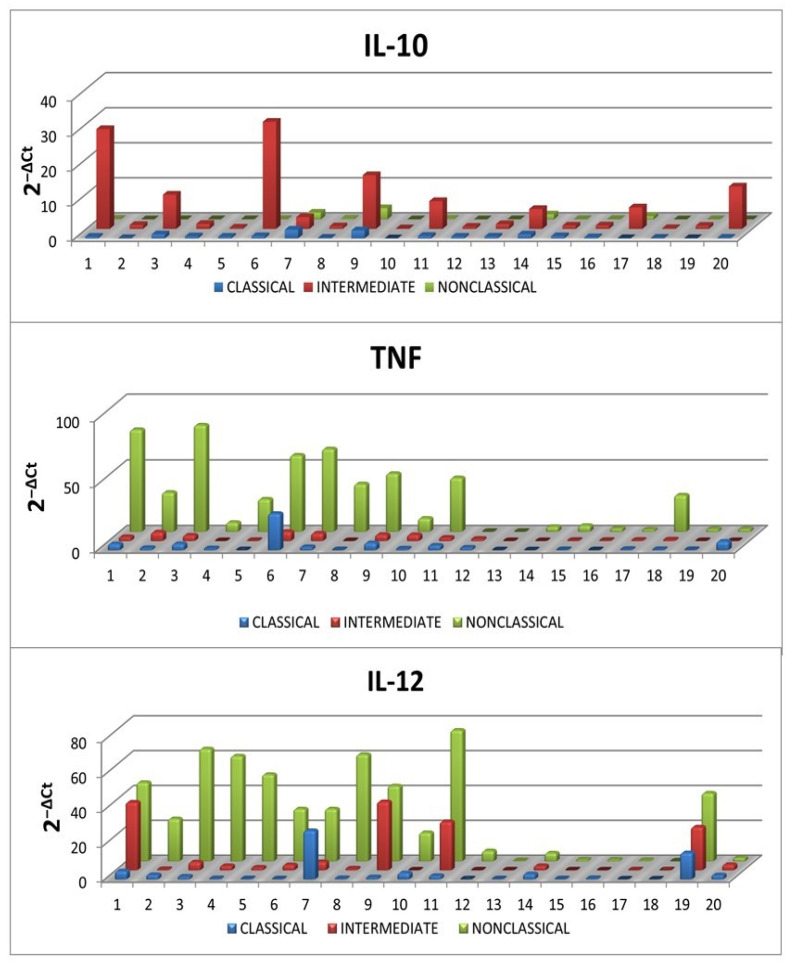
Expression of IL-10, TNF and IL-12 at the mRNA level in classical (CD14^+^CD16^−^SLAN^−^), intermediate (CD14^+^CD16^+^SLAN^−^) and non-classical monocytes (CD14^dim^CD16^+^SLAN^+^) in ex vivo conditions in 20 randomly selected CLL patients.

IL-10, TNF and IL-12 expression levels measured by flow cytometry were confirmed with RT-qPCR. The mRNA levels for IL-10, TNF and IL-12 in classical, intermediate and non-classical monocytes were consistent with the results obtained by flow cytometry (Figure 9).

## 3. Discussion

Individual subpopulations of monocytes have not been the subject of in-depth studies in CLL patients so far. The present study showed that despite the dominance of classical monocytes in CLL patients, the percentage of CD16-positive (intermediate and non-classical) monocytes increased in the course of CLL. As a result, the percentage of both intermediate and non-classical monocytes was significantly higher in CLL patients compared to the control group. A significant increase in the percentage of non-classical monocytes (defined as CD14^+^CD16^++^ cells) in CLL patients was also demonstrated before [14]. Nevertheless, Maffei et al. reported that the percentage of intermediate monocytes was higher in the group of healthy volunteers than in the group of CLL patients [14]. This discrepancy may be attributed to an updated method used in our study, namely with SLAN staining to quantify non-classical monocytes. Maffei et al. identified them only as CD14^low^CD16^high^ cells [14]. Damasceno et al. showed that that the qualification of monocytes to a subpopulation of intermediate monocytes or non-classical monocytes on the basis of the SLAN expression significantly increases the percentage of intermediate monocytes (SLAN-) [15]. Contrary to the current study, Maffei et al. found no correlation between the percentage of individual subpopulations and the stages of CLL, the number of white blood cells or the expression of CD38 or ZAP-70 [14]. Neither did they observe a relationship between the percentage of classical, intermediate or non-classical monocyte subpopulations and gene mutation status for *IgVH*. It should be noted, however, that Maffei et al. conducted their studies on a significantly smaller group of CLL patients (*n* = 26) than in the current study (*n* = 70) [14].

In this study, a negative correlation was found between the percentage of monocytes with the phenotype CD14^dim^CD16^+^SLAN^+^ and lymphocytosis, WBC and the percentage of CD5^+^CD19^+^ leukemic lymphocytes. It can be assumed that this subpopulation may be related to the pathogenesis of CLL. In addition, a significantly higher percentage of non-classical monocytes was observed in CLL patients who, until the end of the follow-up, remained free from disease progression and did not require any therapy. This suggests that a lower percentage of non-classical monocytes may be associated with a more aggressive course of CLL.

It is still disputed whether different subsets of monocytes may express different cytokines [16,17,18]. In our study, the expression of TNF, IL-12 and IL-10 in intermediate, classical and non-classical monocytes was assessed using flow cytometry and RT-qPCR. Non-classical monocytes are the main producers of TNF and IL-12, which seems to confirm the reports of other authors in which it is claimed that SLAN-positive monocytes are pro-inflammatory by nature [17,19,20]. The anti-tumoral properties of SLAN-positive non-classical monocytes suggest their involvement in NK cells’ recruitment and inhibition of Tregs [21].

In contrast, intermediate monocytes seem to mainly produce anti-inflammatory IL-10 [21]. Similarly, Ahmad et al. found that circulating non-classical monocytes have the ability to produce TNF and IL-12 [19]. These observations are also consistent with the reports of Murkherjee et al. who performed cytometric analysis of IL-1β, TNF and IL-10 expression in classical, intermediate and non-classical monocytes in healthy individuals [17]. Nevertheless, both our own results as well as those of Murkherjee et al. differ significantly from the previous report by Cros et al. [16,17]. According to Cros et al., IL-1β and TNF production in response to LPS stimulation is limited to CD14^+^CD16^+^ double-positive monocytes [16]. However, it should be noted that Cros et al. used purified subpopulations of monocytes obtained from the peripheral blood of healthy people for their analysis [16]. However, in our study, intracellular cytokines were measured immediately after the collection of peripheral blood (ex vivo).

Another interesting observation was the increased expression of IL-10 in intermediate monocytes. Those results are again in line with those obtained by Murkherjee et al. [17]. However, they differ from the results of Cros et al. and Wong et al. [16,17,18]. Both teams identified classical monocytes as the main producers of IL-10. In contrast, the CD14^+^CD16^−^ and CD14^+^ CD16^+^ subpopulations showed a moderate synthesis of anti-inflammatory IL-10 [16,18]. It should be noted that the assessment of whole blood (despite LPS stimulation) performed in the study by Murkherjee et al. is closer to physiological conditions (as opposed to the use of isolated cells) [17]. The discrepancy between our research and that presented by Murkherjee et al. and the analysis by Cros et al. and Wong et al. may also be due to the difference in the method used to measure cytokines [16,17,18]. Cros et al. and Wong et al. measured the secreted cytokines with ELISA [16,18]. In this study and the study by Murkherjee et al., the expression of intracellular cytokines was assessed by flow cytometry and also confirmed by RT-qPCR [17]. One of the major limitations of in vitro assessment is the inability to properly mimic the conditions prevailing in vivo. It should also be noted that the intracellular expression of cytokines does not imply the ability to secrete them. In addition, most of the studies reported in the literature involved 18-h [16,18] or 4-h [17] stimulations followed by cytokine analysis. Besides, Ahmad et al. found that SLAN-positive monocytes are particularly sensitive to flow cytometric sorting as they undergo apoptosis rapidly as a result [19].

We showed a significant difference in the percentage of intermediate and non-classical monocytes. The median percentage of non-classical monocytes was significantly higher than the percentage of intermediate monocytes. During acute inflammation such as sepsis, both CD16^+^ subpopulations increase equally with a concomitant decrease in the proportion of classical monocytes [17]. In contrast, in a chronic inflammatory response such as systemic lupus erythematosus (SLE), a subpopulation of non-classical monocytes becomes dominant over intermediate monocytes [17]. However, it is unclear whether the increase in the number of pro-inflammatory non-classical monocytes is a cause or a consequence of the disease. Indeed, an increase in intermediate monocytes may be a factor in differentiating acute and chronic inflammation [17].

In the current study, the percentage of CD14^dim^CD16^+^SLAN^+^ monocytes producing TNF and IL-12 decreased with the stage of CLL and inversely correlated with the expression of the prognostic factors ZAP-70 and CD38. Moreover, it was found to be lower in patients requiring treatment. These observations may suggest that the decrease in the number of CD14^dim^CD16^+^SLAN^+^ is related to the progression of CLL. It is worth noting the frequent coexistence of neoplasms and chronic inflammation, as well as the similar mechanisms of their development [22,23].

## 4. Materials and Methods

### 4.1. Study and Control Groups

The study group consisted of 70 patients with previously untreated chronic lymphocytic leukemia diagnosed in the Department of Haematooncology and Bone Marrow Transplantation, Medical University of Lublin, according to the criteria of the International Workshop on Chronic Lymphocytic Leukemia (IWCLL) [24]. The ages of the patients ranged from 37 to 85 years (median: 65).

The study group included 44 males (63%) and 26 females (37%) (M:F-1.69). Patients were divided into 3 groups based on the Rai classification [25,26]: the low-risk group (stage 0 by Rai)—28 patients; the intermediate-risk group (stage I/II by Rai)—34 patients; and the high-risk group (stage III/IV according to Rai)—8 patients.

The control group consisted of 35 healthy volunteers (20 men, 15 women). The ages of the donors ranged from 35 to 82 years (median: 58).

Approximately 5 mL of peripheral blood was collected from CLL patients and control group subjects into EDTA-3K coated tubes (S-Monovette, SARSTEDT, Numbrecht, Germany). The peripheral blood sample was used for isolation of peripheral blood mononuclear cells (PBMC).

The time of observation of CLL patients ranged from 0.5 to 48 months (median: 38 months). During the follow-up period, 24 patients required therapy. Characteristics based on clinical and laboratory aspects of the group of assessed CLL patients are presented in Table 1.

### 4.2. Ethical Approval

The study was conducted according to the guidelines of the Declaration of Helsinki, and approved by the Bioethics Committee of the Medical University of Lublin (No. KE-0254/49/2016, date of approval: 25 February 2016; KE-0254/239/2017, date of approval: 28 September 2017). All patients gave written informed consent to participate in the research.

### 4.3. Isolation of Peripheral Blood Mononuclear Cells (PBMC)

Peripheral blood samples, collected in EDTA-3K coated tubes, were used for mononuclear cells’ separation by density gradient centrifugation on Gradisol L (Cat No.: 9003.1, Aqua-Med, Łódź, Poland) for 20 min at 700× *g* at room temperature. A layer of PBMCs was harvested as the interphase between Gradisol L and the diluted plasma. Isolated PBMCs were washed twice in PBS, and then their numbers (in a Neubauer chamber) and viability were assessed. Viability below 95% disqualified PBMCs from further studies.

### 4.4. Flow Cytometry Analysis of Cell Viability

BD Horizon Fixable Viability Stain 510 (FVS510) (Cat No.: 564406; BD Biosciences, Franklin Lakes, NJ, USA) was used in order to distinguish dead cells, based on multicolor flow cytometry. Suspensions of cells with a density of 1 × 10^6^ cells were incubated for 15 min at room temperature in the darkness. As the next step, cells were washed twice (Stain Buffer; Cat No.: 554657, BD Biosciences) and stained with monoclonal antibodies.

### 4.5. Flow Cytometry Analysis of Subpopulations of Monocytes

After labeling cells viability dye (as described in point 4.4), 1 × 10^6^ PBMCs were incubated with monoclonal antibodies: Mouse Anti-Human CD14 V450 (clone: MφP9, Cat No.: 560349, BD Biosciences) and Mouse Anti-Human CD16 FITC (clone 3G8, Cat No.: 555406, BD Biosciences). Prepared cells were incubated for 10 min at room temperature, after which the incubation with Mouse Anti-Human SLAN APC (M-DC8) (clone: DD-1; Cat No.: 130-119-865, Miltenyi Biotec, Bergisch Gladbach, NRW, Germany) was continued at 2–8 °C for another 10 min. Then, cells were washed 2 times with PBS (5 min; 700× *g*) and subjected to cytometric analysis using a FACSCanto II flow cytometer with FACSDiva Software (BD Biosciences, Franklin Lakes, NJ, USA). Kaluza 2.1.1 (Beckman Coulter, Miami, FL, USA) was used to analyze and graphically present the collected data.

### 4.6. Analysis of Intracellular Expression TNF, IL-12, IL-10 in SLAN-Positive and SLAN-Negative Monocytes

Cytokine expression was assessed ex vivo directly after taking blood samples from patients. As described above, subpopulations of classical intermediate and nonclassical monocytes were identified by monoclonal antibodies: anti-CD14 V450 (clone MφP9, Cat No.: 560349, BD Biosciences), anti-CD16 FITC (clone 3G8, Cat No.: 555406, BD Biosciences) and anti-SLAN APC (M-DC8) (clone: DD-1; Cat No.: 130-119-865, Miltenyi Biotec). First, dead cells were stained with FVS510. Then, cells were stained using monoclonal antibodies against surface markers, fixed (using Cytofix/Cytoperm) and permeabilized (using Perm/Wash) (Cat No.: 554714, BD Biosciences). Cells prepared in this way were incubated with monoclonal antibodies: Mouse Anti-Human TNF PE (clone: MAb11, Cat.No 559321, BD Biosciences), Mouse Anti-Human IL-12 PE (p40/p70) (clone: C11.5, Cat No. 559329, BD Biosciences) and Rat Anti-Human IL-10 PE (clone: JES3-19F1, Cat. No. 559330, BD Biosciences). Then, cells were incubated for 20 min (in the darkness, room temperature) and finally washed twice (5 min, 700× *g*) with a buffered saline solution devoid of Ca^2+^ and Mg^2+^ ions (PBS). At the end of the procedure, samples were analyzed on a FACSCanto II flow cytometer. The fluorescence minus one control (fluorescence-minus-one, FMO) was used to set proper gates.

### 4.7. SLAN-Positive and SLAN-Negative Monocytes Sorting for RT-qPCR

Monocyte subpopulations, after staining with the anti-CD14, anti-CD16 and anti-SLAN antibodies, were sorted using BD FACSAria II (BD Biosciences). Anti-CD14 PE (clone MφP9, Cat No. 345785, BD Biosciences) and anti-CD16 FITC (clone 3G8, Cat No. 555406, BD Biosciences) and anti-SLAN APC (M-DC8) (clone DD-1, Cat No. 130-119-865, Miltenyi Biotec) monoclonal antibodies were used to identify individual subpopulations. CD14^+^CD16^−^SLAN^−^, CD14^+^CD16^+^SLAN^−^ and CD14^dim^CD16^+^SLAN^+^ subpopulations were collected with a purity above 95%. The purity of each sort was assessed shortly afterwards. RNA was isolated from classical, intermediate and nonclassical monocytes cleared in the sorting process. Total RNA was isolated using the QIAamp RNA Blood Mini Kit (Cat No.: 52304; Qiagen, Inc., Valencia, CA, USA).

### 4.8. RT-qPCR for TNF, IL-12 and IL-10

Sorted CD14^+^CD16^−^SLAN, CD14^+^CD16^+^SLAN^−^ and CD14^dim^CD16^+^SLAN^+^ monocytes were analyzed for TNF, IL-12 and IL-10 mRNA expression in 20 randomly selected CLL patients’ samples. The Quantitative Reverse Transcription Polymerase Chain Reaction (RT-qPCR) method was performed as previously described [27]. All molecular tests were based on TaqMan Gene Expression Assays (Thermo Fisher Scientific, Applied Biosystems, Inc., Waltham, MA, USA) using the following assays: Hs00174128_m1 probe for TNF-alpha, Hs01073447_m1 probe for IL-12A and Hs00961622_m1 probe for IL10. TaqMan Gene Expression Master Mix (Cat No.: 4369016) was used for all reactions. In this study, β-actin (Human ACTB (Beta Actin) Endogenous Control, Cat No.: 4310881E, Thermo Fisher Scientific, Applied Biosystems, Inc., Waltham, MA, USA) was used for normalization. Data are presented as 2^−ΔCq^.

### 4.9. Statistical Analysis

The statistical analysis was carried out using Statistica 13.0 software (StatSoft, Cracow, Poland). The GraphPad software version 5.0 (GraphPad Software, San Diego, CA, USA) was used for the graphic presentation of the obtained results. For data description, the median and interquartile range (IQR: 25% percentile and 75% percentile) were used. The Mann–Whitney U test was used to compare two independent groups, Wilcoxon’s pairwise test was used to compare related variables within groups, and Spearman’s rank correlation test was used to assess the dependence between the variables. The ROC (Receiver Operating Curve) graphs were used to determine the optimal cut-off points that could be a prognostic factor for the time-to-treatment-initiation. The Kaplan–Meier analysis was used to assess the probability of the need to start cytostatic treatment. The log-rank test was used to assess the difference in time from disease diagnosis to time-to-treatment (TTT) between groups. Cox regression analysis was constructed to determine the hazard ratio (HR). The level of significance was set at *p*-value ≤ 0.05.

## 5. Conclusions

In conclusion, this paper presents the characteristics of individual subpopulations of monocytes and their relation to the clinical course of CLL. Our results indicate that the SLAN-negative population of monocytes has anti-inflammatory characteristics, while the SLAN-positive population has anti-tumor characteristics in the CLL microenvironment.

## Figures and Tables

**Table 1:** Clinical and laboratory characteristic of CLL patients in groups with high and low percentages of SLAN-positive non-classical monocytes (nMo).

Parameter		All Patients	nMo^high^ (>6.37%)	nMo^low^ (<6.37%)
	No. of patients	70	37	33
Sex	Female *	26 (37.14)	9 (24.32)	17 (51.52)
	Male *	44 (62.86)	28 (75.68)	16 (48.48)
Risk groups	Low-risk (Stage 0) *	28 (40.00)	17 (45.95)	11 (33.33)
	Intermediate-risk (Stage I–II) *	34 (48.57)	15 (40.54)	19 (57.58)
	High-risk (Stages III–IV) *	8 (11.43)	5 (13.51)	3 (9.09)
ZAP-70 (cut off 20%) ^a^	ZAP-70-positive *	27 (38.57)	12 (32.43)	15 (45.45)
	ZAP-70-negative *	43 (61.43)	25 (67.57)	18 (54.55)
CD38 (cut off 30%) ^b^	CD38-positive *	25 (35.71)	13 (35.14)	12 (36.36)
	CD38-negative *	45 (64.29)	24 (64.86)	21 (63.64)
Cytogenetic aberrations	del(11q22.3) or/and del(17p13.1) *	14 (20.59)	8 (21.62)	6 (18.18)
	Without del(11q22.3) or/and del(17p13.1) *	56 (79.41)	29 (78.38)	27 (81.82)
	Patients requiring treatment *	24 (34.29)	8 (21.62)	16 (44.48)
	Untreated patients *	46 (65.71)	29 (78.38)	17 (51.52)
WBC count (G/L) #		26.03 (10.11–194.54)	24.39 (10.11–114.84) ^$^	35.18 (13.39–194.54) ^$^
LYMPH (G/1) #		19.38 (5.51–181.09)	18.60 (5.51–109.55) ^$^	30.87 (9.57–181.09) ^$^
LDH (IU/L) #		373 (231–618)	356 (266–533)	383 (231–68)
Hemoglobin (g/dL) #		13.95 (8.00–17.20)	14.30 (11.30–16.10)	13.70 (8.10–17.20)
PLT (G/L) #		189.00 (49.00–525.00)	189 (49–525)	180 (90–399)
β2M (mg/dl) #		2.35 (1.36–12.37)	2.29 (1.45–6.04)	2.54 (1.36–12.37)
% cells CD19^+^/CD5^+^/ZAP-70^+^ ^a^ #		15.84 (0.21–59.05)	12.20 (1.58–59.05) ^$^	17.15 (0.21–56.21) ^$^
% cells CD19^+^/CD5^+^/CD38^+^ ^b^ #		10.18 (0.22–80.90)	10.11 (0.66–80.90)	9.92 (0.22–79.98)

* number (percentages); # median (minimum-maximum); nMo, SLAN-positive non-classical monocytes; WBC, white blood cells; LYMPH, absolute lymphocyte count; LDH, lactate dehydrogenase; β2M, β2-microglobulin; PLT, absolute platelet count. ^a^ Patients with percentage of CD19^+^CD5^+^ cells with intracellular ZAP-70 expression ≥20% were classified as ZAP-70-positive. ^b^ Patients with percentage of lymphocytes CD19^+^CD5^+^CD38^+^ ≥ 30% were classified as CD38-positive. ^$^ *p* < 0.01.

**Table 2:** Univariate and multivariate analysis for time-to-treatment.

Univariate				Multivariate	
Variable	Median TTT (Months)	HR (95% CI)	*p*	HR (95% CI)	*p*
ZAP-70					
≥20%	37	2.89 (1.27–6.57)	**<0.01**	1.23 (0.35–4.52)	**0.79**
<20%	30				
CD38					
≥30%	37	2.81 (1.26–6.22)	**<0.01**	1.88 (0.56–6,37)	0.308
<30%	29				
β2M					
≥3.5 mg/dL	39	4.94 (2.06–11.86)	**<0.001**	5.83 (2.13–15.926)	**<0.001**
<3.5 mg/dL	29				
del(17p13.1) or del(11q22.3)					
Positive	34	0,82 (0.18–3.56)	0.392		
Negative	47				
nMo^low^					
≥6.37%	35	3.12 (1.33–7.34)	**<0.01**	2.26 (0.98–5.23)	0.055
>6.37%	39				
Age					
≥65 years	44	0.96 (0.43–2.16)	0.360		
<65 years	47				

TTT, time-to-treatment; β2M, β2 microglobulin; nMo, SLAN-positive non-classical monocytes; HR, hazard ratio; 95% CI: 95% confidence interval. Only variables with *p* < 0.05 in the univariate analysis were added to the multivariate analysis.

## Data Availability

The data presented in this study are available within the article. Other data that support the findings of this study are available upon request from the corresponding author.
